# Molecular Comparison and Evolutionary Analyses of VP1 Nucleotide Sequences of New African Human Enterovirus 71 Isolates Reveal a Wide Genetic Diversity

**DOI:** 10.1371/journal.pone.0090624

**Published:** 2014-03-05

**Authors:** Maël Bessaud, Richter Razafindratsimandresy, Antoine Nougairède, Marie-Line Joffret, Jagadish M. Deshpande, Audrey Dubot-Pérès, Jean-Michel Héraud, Xavier de Lamballerie, Francis Delpeyroux, Jean-Luc Bailly

**Affiliations:** 1 Aix Marseille Univ, IRD French Institute of Research for Development, EHESP French School of Public Health, UMR_D 190 “Emergence des Pathologies Virales”, Marseille, France; 2 Institut Pasteur de Madagascar, Unité de Virologie, Antananarivo, Madagascar; 3 Institut Pasteur, Unité de biologie des virus entériques, Paris, France; 4 INSERM U994, Paris, France; 5 Enterovirus Research Centre, Mumbai, India; 6 Lao-Oxford-Mahosot Hospital-Wellcome Trust Research Unit, Mahosot Hospital, Vientiane, Lao PDR; 7 Centre for Clinical Vaccinology and Tropical Medicine, University of Oxford, Oxford, United Kingdom; 8 Clermont Université, Université d'Auvergne, EPIE EA4843, Clermont-Ferrand, France; University of Rome Tor Vergata, Italy

## Abstract

Most circulating strains of Human enterovirus 71 (EV-A71) have been classified primarily into three genogroups (A to C) on the basis of genetic divergence between the 1D gene, which encodes the VP1 capsid protein. The aim of the present study was to provide further insights into the diversity of the EV-A71 genogroups following the recent description of highly divergent isolates, in particular those from African countries, including Madagascar. We classified recent EV-A71 isolates by a large comparison of 3,346 VP1 nucleotidic sequences collected from GenBank. Analysis of genetic distances and phylogenetic investigations indicated that some recently-reported isolates did not fall into the genogroups A-C and clustered into three additional genogroups, including one Indian genogroup (genogroup D) and 2 African ones (E and F). Our Bayesian phylogenetic analysis provided consistent data showing that the genogroup D isolates share a recent common ancestor with the members of genogroup E, while the isolates of genogroup F evolved from a recent common ancestor shared with the members of the genogroup B. Our results reveal the wide diversity that exists among EV-A71 isolates and suggest that the number of circulating genogroups is probably underestimated, particularly in developing countries where EV-A71 epidemiology has been poorly studied.

## Introduction

Human enterovirus 71 (EV-A71) is a member of the *Enterovirus A* species, genus *Enterovirus*, family *Picornaviridae*. EV-A71 circulates worldwide and mainly affects children. A dermotropic virus, it is one of the etiologic agents of hand-foot-and mouth disease, a common and self-limiting syndrome in children characterized by fever, papulovesicular rash on the palms and the soles, and oral ulcers [1]. During outbreaks in the 1970s and 1980s, it was infrequently associated with neurological manifestations including aseptic meningitis and polio-like acute flaccid paralysis [2], [3]. Since the outbreaks of 1997 and 1998 in Malaysia and Taiwan, respectively, EV-A71 has been responsible for severe manifestations throughout the Asia-Pacific region, in particular in China [4]. Of greatest concern among the consequences is brain stem encephalitis and, in some children, pulmonary edema and hemorrhage with subsequent cardiopulmonary collapse and shock. Specific prophylaxis and treatments are not currently available against EV-A71 infection but different vaccines were recently tested in phase 2 and 3 trials [5], [6].

The virus particles have an icosahedral shape, are not enveloped, and contain a single positive RNA genome of about 7,400 nucleotides (nt). The genome contains two non-coding regions that flank a single open reading frame. The viral polyprotein is processed by proteolytic cleavages to give rise to the functional proteins. Its N-terminal part contains the four structural proteins (VP1 to VP4) that are assembled to form the virion and its C-terminal part contains the non-structural proteins.

Historically, typing of enteroviruses consisted in identifying the serotype by serological neutralization. Because neutralization methods are labour-intensive, they have been replaced by molecular methods based on the correlation between the serotype and the genotype determined by sequencing of the structural region of the genome [7], [8], [9]. This correlation relies on the immunologically-driven evolution of structural genes, since the capsid proteins bear the major viral epitopes recognized by neutralizing antibodies. The most commonly-used method of enterovirus genotyping targets the 1D gene (∼900 nt in length) that encodes the VP1 capsid protein, which contains the major neutralizating epitopes. The VP1 gene sequences of circulating strains are compared with the sequences of prototype strains (a list is available through the Picornaviridae Study Group website at http://www.picornaviridae.com/enterovirus/enterovirus.htm); homotypic viruses usually share more than 75% nt similarity and 85% amino acid (aa) identity [10].

The first phylogenetic analysis of EV-A71 based on VP1 sequences identified three distinct genogroups designated A, B and C [11]. Genogroup A includes the EV-A71 prototype strain BrCr, which was isolated in California in 1969; for decades thereafter, no other circulating strains were observed in this genogroup. However, virus isolates that displayed a puzzling genetic relatedness with the BrCr strain were collected in China in the 2000s and 2010s [12], [13]. Genogroups B and C contain hundreds of viruses isolated worldwide, which were grouped into subgenogroups B0–B5 and C1–C5, on the basis of phylogenetic relationships of nt sequences [1]. In 2003, a putative fourth genogroup was identified in India by partial sequencing of the VP1 encoding gene of one isolate [14]. More recently, a probable fifth genogroup was identified in sub-Saharan Africa [15], [16].

The aim of this study was to provide further insights into the genetic diversity of EV-A71 genogroups by the description of recently identified, highly divergent isolates, in particular those from African countries, including two new isolates from Madagascar. We considered a large dataset of VP1 sequences and assigned them to six distinct genogroups after comprehensive molecular and phylogenetic analyses of the genetic diversity within the EV-A71 type. We also estimated the evolutionary history of all genogroups with Bayesian coalescent-based statistical methods and found in particular that the tentative new genogroup described herein emerged in the mid 1990s.

## Methods

### Construction of the VP1 sequence dataset

Three thousand four hundred fifty seven sequences were retrieved from GenBank following a search with the key words ‘enterovirus 71’, ‘VP1’, ‘capsid’ and/or ‘complete genome’ and were then subjected to manual checking. Partial sequences (<891 nt) and those comprising stop codons and frame shift mutations were rejected. Manual editing and exploratory alignments were done with CLC Main Genomics 6 software (CLC bio). As redundant sequences were not discarded, the final dataset may contain identical sequences from a single viral isolate but reported under different accession numbers.

In addition, the partial VP1 sequence of isolate R13223 reported earlier [14] was updated after sequencing of the complete gene (GenBank accession number AY179600) and included in our analyses.

The final dataset also contained two hitherto unpublished VP1 sequences of two field isolates collected in 2004 and 2011 (GenBank accession numbers HG421068 and HG421069, respectively) from stool specimens of two healthy children living in the district of Taolagnaro, in southern Madagascar (Razafindratsimandresy & Delpeyroux, unpublished data).

### Sequence analysis by distance methods

Phylograms based on full-length VP1 sequences were constructed with the MEGA 5 software [17]. The evolutionary distances were computed with different algorithms (Juke-Cantor model, Maximum Composite Likelihood model, Kimura 2-parameter model) and the corresponding trees were drawn with the Neighbor-joining method with 500 bootstrap replicates to estimate node consistency.

For the purpose of molecular comparisons and Bayesian evolutionary analyses (see below), a subset of 59 sequences representing all genogroups, subgenogroups and outlier lineages of EV-A71 was selected by exploratory analyses of the complete sequence dataset (Table S1).

### Recombination analysis

Recombination was analyzed with the SimPlot software [18]. The algorithm allowed the calculation of pairwise similarity between all sequences in a multiple sequence alignment. We calculated the pairwise similarity between the sequences using a 100-nt wide window moving along the alignment through steps of 20 nt.

### Bayesian phylogenetic analyses

The evolutionary rate, divergence time, and demographic history were co-estimated in a Bayesian statistical framework using a Markov Chain Monte Carlo (MCMC) approach implemented in the BEAST program version 1.7.5 ([19], [20]; http://beast.bio.ed.ac.uk). Two substitution models were compared: the general-time-reversible (GTR) model with an invariant class of nt substitution and a gamma distribution of substitution rates and the SRD06 model [21]. As coalescent priors, the Bayesian skyline [22] and Gaussian Markov random fields (GMRF) Bayesian skyride [23] models were compared under relaxed molecular clock models. Models were compared with a Bayesian method of model selection that uses Bayes factors [24]. The Bayes factor indicates relative superiority of competing models by evaluating the ratio of their marginal likelihoods. The marginal likelihood distributions were estimated with the method of Suchard et al. [25] implemented in the tracer v.1.5.0 program [20] available at http://tree.bio.ed.ac.uk/software/tracer/. MCMC analyses were run for 40 million generations, sampling a tree every 2,000 steps and the first 10% were discarded as burn-in. MCMC convergence and the effective sample sizes (ESS) estimates were checked with Tracer v1.5.0. The trees sampled during the MCMC process were summarized in a maximum clade credibility (MCC) tree, as estimated with the TreeAnnotator program (http://beast.bio.ed.ac.uk/TreeAnnotator), and produced with the FigTree program (http://tree.bio.ed.ac.uk/software/figtree). Statistical support for the tree nodes was assessed by a posterior probability (pp) value. Time to the most recent common ancestor (TMRCA) at each node in the phylogeny was calculated from the height value in the MCC tree. Statistical uncertainty in the TMRCA calculations was estimated as a 95% highest posterior density (HPD) interval. The reproducibility of all phylogenetic trees was tested by multiple BEAST runs.

## Results

### Analysis of the VP1 sequences of the two EV-A71 isolates from Madagascar

The two original VP1 sequences determined in this study from the MAD-72341-04 and MAD-3126-11 isolates shared substantial nt (91.6%) and aa (96.9%) similarities. They were assigned unambiguously to the EV-A71 type, on the basis of >80.1% nt similarity and >93.6% aa identity with the BrCr prototype (Table 1). However, an exploratory phylogenetic analysis that included these two sequences and a few VP1 sequences representative of the genogroups A to E suggested that they clustered together into a single clade clearly distinct from the phylogenetic genogroups already described (data not shown). This new clade was tentatively called “genogroup F”.

**Table 1:** Similarity within the EV-A71 genogroups through comparison of the full-length VP1 gene sequences.

		Within genogroup similarity (%)		Similarity with the BrCr prototype sequence (%)	
Genogroup	No of sequences	nt	aa	nt	aa
A	15	>97.3	>94.9	-	-
B	763	>84.6	>95.9	>79.9	>92.2
C	2,563	>83.9	>94.2	>80.2	>91.9
D	1	-	-	81.3	95.2
E	2	89.5	96.9	>79.9	>93.6
F	2	91.6	96.9	>80.1	>93.6

**Table 2:** Selection of an evolutionary model for the EV-A71 VP1 sequence dataset, through comparison of marginal likelihood.

		Log marginal likelihood estimate	
Molecular clock model	Demographical model	Path sampling method	Stepping-stone method
UCED	Bayesian skyline	−8663.3078	−8663.6138
UCED	GMRF Bayesian skyride	−8666.6196	−8667.0055
UCLN	Bayesian skyline	−8647.8394	−8648.2044
UCLN	GMRF Bayesian skyride	−8639.4162	−8639.5518

The analyses were done with the SRD06 substitution model and a relaxed molecular clock model implementing either an uncorrelated exponential (UCED) or an uncorrelated lognormal (UCLN) distribution of clock rates among the lineages. The molecular clock and demographic models were determined by calculating the marginal likelihoods of the data conditional on all the evolutionary and demographic model parameters. The highest log marginal likelihood values indicate the best fit model.

### Genetic relationships between the EV-A71 VP1 gene sequences

A comprehensive phylogenetic analysis was performed to investigate the genetic relationships of the two original Madagascan sequences and to classify them consistently among the current EV-A71 strains. We analyzed a large dataset of complete VP1sequences available on public databases. The final dataset contained 3,346 sequences, including the two original sequences of isolates MAD-72341-04 and MAD-3126-11 from Madagascar and the updated sequence of isolate R13223 from India. All the sequences comprised 891 nt and encoded a protein of 297 aa residues. The nt and aa alignments displayed a substantial proportion of positions conserved among all the sequences, 277/891 (31%) and 74/297 (25%) respectively.

**Figure 1:**
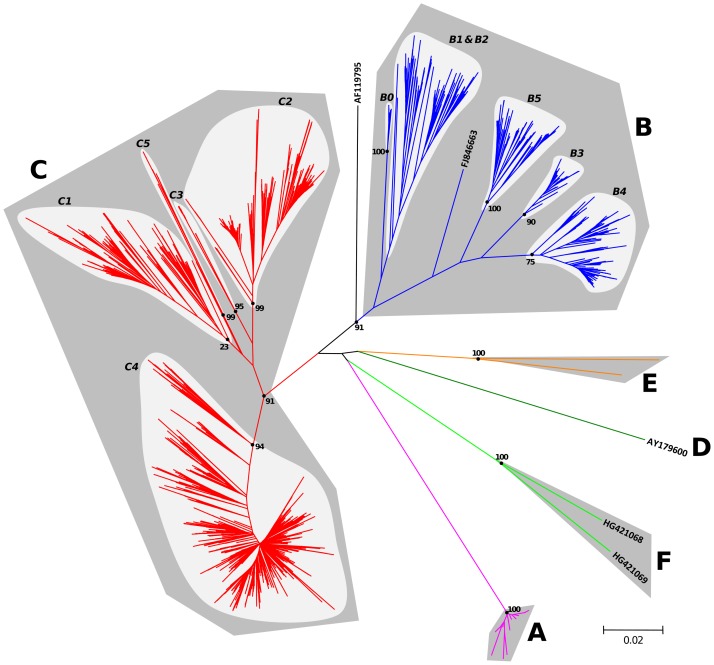
Phylogenetic relationships between EV-A71 full-length VP1 sequences inferred using the Neighbor-Joining method. The evolutionary distances were computed using the Kimura 2-parameter method. The length of the branches is proportional to the number of nt divergence. The percent of bootstrap replicates is indicated for the main nodes.

The genetic relationships between the nt sequences were investigated with the Neighbor-Joining method by using three different distance models. The tree topologies estimated with the different models displayed only minor differences (data not shown). The tree estimated with the Kimura 2-parameter model is shown in Figure 1.

The clade corresponding to genogroup A (bootstrap value of 100%) included the BrCr prototype sequence and 14 sequences determined in viruses isolated in China in 2008 and 2009 [12], [13]. The Chinese strains displayed together an overall nt difference ≤2.7% (Table 1). Additionally, they featured a close relatedness with BrCr, in spite of the four decades that separate their emergence and the isolation of BrCr.

The 763 (22%) sequences corresponding to genogroup B shared ≥84.6% nt similarity and ≥95.5% aa identity (Table 1). The genogroup B sequences segregated into distinct clusters corresponding to the reported subgenogroups B0 to B5. Sub-clusters B0, B3 and B5 were supported by high bootstrap values (≥90%) in contrast to subcluster B4 (75%). The unpublished sequence with the assigned accession number FJ846663 clustered separately from the other B subgenogroups (Figure 1).

The major clade in the EV-A71 tree (Figure 1) comprised 2,563 (75%) sequences assigned to genogroup C and was supported by a bootstrap value of 91%. The sequences exhibited ≥83.9% nt similarity and ≥91.2% aa identity (Table 1). The sequences of this genogroup segregated into distinct clusters corresponding to the reported subgenogroups C1 to C5. Subgenogroups C2, C3, C4 and C5 were supported by high bootstrap values (≥94%). The low bootstrap value (23%) that supports the C1 cluster could be related to few phylogenetic features shared by a small number of C1 sequences and sequences of the other subgenogroups C2, C3, and C5, thus resulting in uncertainty in estimation of the C1 root. This could be due to recombination events between viruses belonging to C1 genogroup and viruses belonging to other C subgenogroups or, most probably, to convergent evolution or parallel nucleotide substitutions at similar sites among the different subgenogroups.

One previously reported sequence with the accession number AF119795 had been assigned earlier to the genogroup C [26]. In our phylogenetic analysis, it was actually more closely related to the genogroup B but clustered separately from the other subgenogroups (Figure 1). The AF119795 sequence was compared with sequences selected as representative of the EV-A71 genogroups to determine the pairwise nt similarity by a scanning analysis along the sequence alignment (Figure S1). The AF119795 sequence displayed a high similarity (range 93–98%) with sequences of genogroup B only in a large central part of the VP1 gene (approximate nt positions 120 and 660). In the 5′ and 3′ parts of the VP1 gene, the AF119795 sequence displayed high similarity with genogroup C sequences. This suggests a recombinant origin of the AF119795 sequence via a possible intratypic recombination event between ancestor sequences belonging to genogroups B and C. However, this possibly should be considered cautiously: although already documented [27], [28], [29], [30], intratypic and intertypic recombination events within the capsid-encoding region are believed to be rare because of structural constraints on full assembly of the capsid [31]. Hence, we cannot exclude the possibility that the AF119795 sequence arose as a result of gene amplification and sequencing artifacts caused by a mixture of viruses of genogroups B and group C.

A distinct lineage included the single genogroup D sequence, from a virus collected in India in 2001 [14]. In a previous study, 12 partial VP1 sequences were assigned to a tentative genogroup F [32]. In our analysis, these sequences displayed close genetic relationships with the R13223 sequence (>88.7% nt homology, >95.6% aa identity), which strongly suggests that these partial sequences should be assigned to genogroup D (Figure S2).

Two sequences determined in viruses collected in Central African Republic and Cameroon [15], [16] displayed a nt similarity of 85.9% (96.9% aa identity), and together formed a distinct genogroup supported by a bootstrap value of 100% and previously designated genogroup E (Figure 1).

The phylogenetic tree confirmed that the two Madagascan sequences determined in this work clustered separately from the genogroups A–E in a single clade supported by a bootstrap value of 100% (Figure 1). This clade was tentatively called ‘genogroup F’. It contained no other sequence present in our dataset and BLAST searches conducted in public databases failed to identify any complete or partial VP1 sequence closely related to the two sequences belonging to genogroup F.

Interestingly, 20 partial VP1 sequences that were reported recently from viruses collected in India [32] did not fall into any of the six genogroups A to F (Figure S2). This strongly points to the existence of additional genogroups. However, as these observations were based on only partial VP1 sequences (some of which were <400 nt in length), they will need to be confirmed in the future by analyzing longer genomic sequences.

### Molecular divergence between the enterovirus 71 genogroups

**Figure 2:**
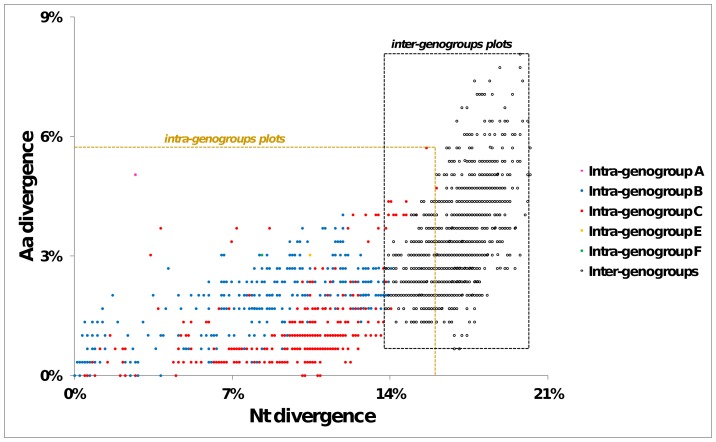
Molecular differentiation of the EV-A71 genogroups. The percentages of nucleotide and amino acid divergences were calculated in pairwise comparisons of 59 sequences selected as representative of the EV-A71 genogroups and subgenogroups. A scatter plot was produced with the divergence values to determine a threshold between intra-and inter-genogroup differences.

The molecular divergence between the different genogroups and subgenogroups was analyzed with a set of 59 VP1 sequences (Table S1) selected on phylogenetic grounds to represent the overall genetic diversity of type EV-A71. The results of all pairwise comparisons between sequences are represented as a scatter plot (Figure 2). The divergence between sequences assigned within a same genogroup was in the range of 0–16.0% nt differences and 0–5.7% aa differences (Table S2). The divergence between sequences assigned to different genogroups was in the range of 13.8–20.2% nt differences and 0.6–8.1% aa differences (Table S2).

There was an overlap of 2.2% between the ranges of intra-genogroup and inter-genogroup nt divergence, but divergence values above a threshold of 16.0% indicated that two EV-A71 sequences belonged to distinct genogroups. Similarly, below a threshold of 13.8% nt differences, any two sequences were assigned to a same genogroup. Accordingly, a nt divergence within the range of 13.8–16.0% does not permit to determine whether two sequences belong to the same genogroups or not.

Finally, the percentage of aa differences was not a reliable feature for differentiating two genogroups since there was a wide overlap between the ranges of intra- and inter-genogroup divergence.

### Evolutionary origin of the EV-A71 strains from Madagascar

The evolutionary history of the EV-A71 genogroups was investigated with the sample of 59 sequences (Table S1). Among the evolutionary models analyzed, those including the GTR substitution model fit the data better than those including the SRD06 model (Bayes factors >40). There was no statistical difference (Bayes factors <5) between the two distributions of evolutionary rates analysed in the relaxed-clock model (uncorrelated exponential distribution and uncorrelated log normal distribution of clock rates, uced and ucln respectively) nor between the different demographical models. The substitution rates estimated with the different evolutionary models were in the range of 3.60×10^−3^ to 5.345×10^−3^ nt substitutions per site per year (Figure S3). The 95% HPD intervals of the evolutionary rate estimates displayed substantial overlap, which indicated significant robustness to the various estimates. However, the substitution rate values calculated with the GTR model were lower than those estimated with the SRD06 model.

The maximum clade credibility tree summarizing the posterior tree distribution estimated with the VP1 sequence sample was inferred with the evolutionary model based on the GTR substitution model, an uced distribution of clock rates, and the Bayesian skyline plot demographical model.

**Figure 3:**
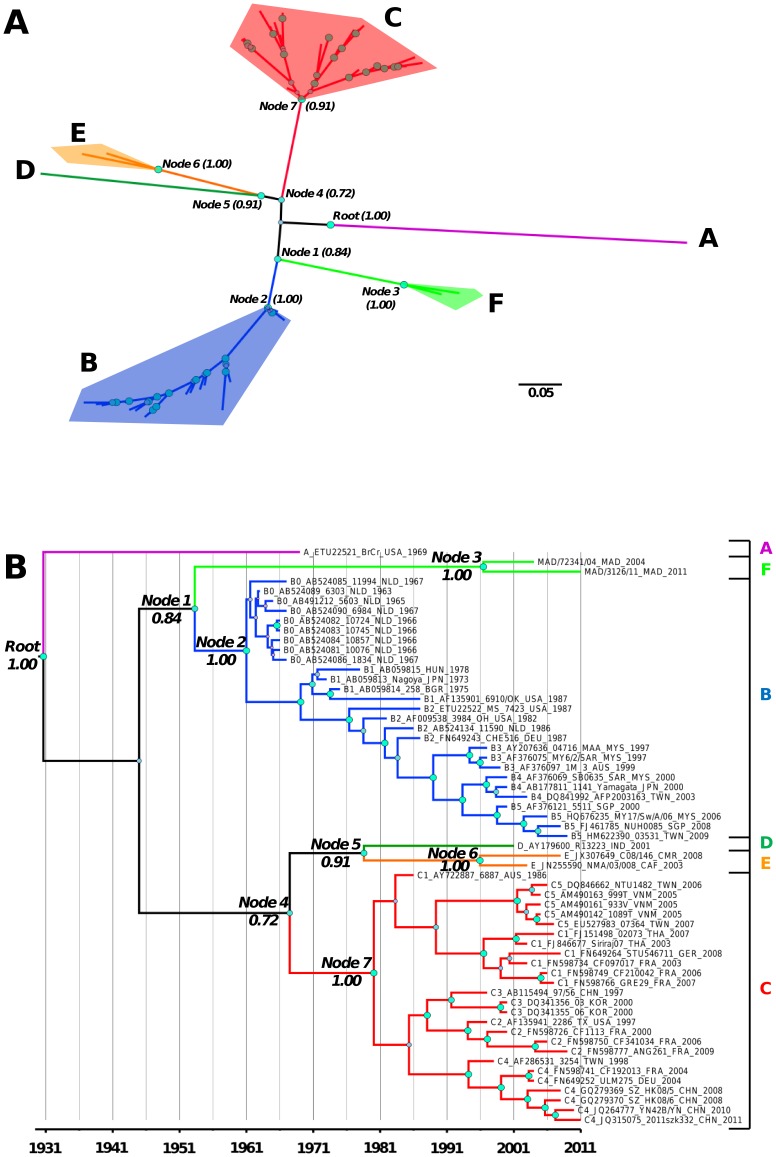
Bayesian maximum clade credibility phylogram (A) and chronogram (B) inferred with the 1D gene encoding the VP1 capsid protein, depicting the phylogeny of EV-A71. The trees were estimated with general time reversible nucleotide substitution model, an uncorrelated exponential clock model, and the Bayesian skyline as a tree prior. The posterior probability of the eight nodes used to compare different evolutionary models (see also Figures 4 and 3) are indicated. The posterior probability (pp) are indicated for the main nodes.

The Bayesian phylogram topology (Figure 3A) was congruent with the topology previously observed in the Neighbor-Joining tree (Figure 1), with six different genogroups supported by pp>0.91. Notably, the newly described genogroups E and F were supported by pp of 1.

The Bayesian trees (Figures 3A and 3B) showed evidence of two major partitions identified by the ancestral nodes 1 and 4, and of distinct clades (pp>0.9) characterized by nodes 2, 3, and 5–7. A third tree partition was represented by the clade of genogroup A. The partition depicted by node 1 included a clade (node 3, pp = 1) containing the two viruses collected in Madagascar, which clearly indicates that this cluster should be considered as a new genogroup. In this partition, the clade corresponding to genogroup B (node 2, pp = 1) provided evidence of a strong temporal clustering of sequences. The second major tree partition was characterized by a radiation of three clades from node 4, those of genogroups C (node 7, pp = 1), D (node 5, pp = 0.91), and E (node 6, pp = 1).

**Figure 4:**
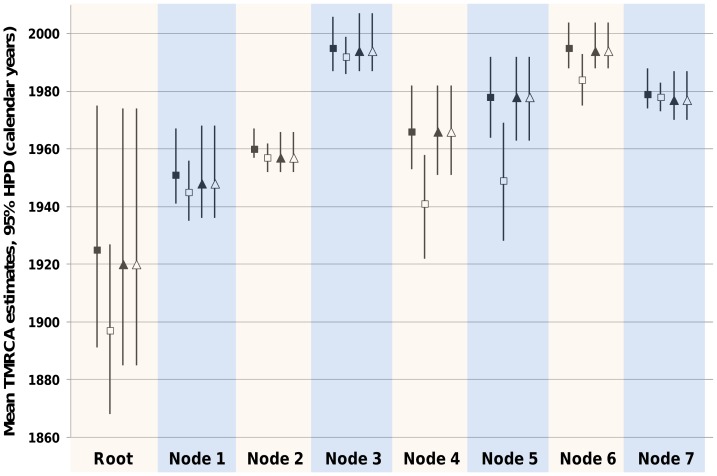
Time to the most recent common ancestor (TMRCA) calculated with different models for major nodes of interest within the EV-A71 phylogeny (see also Figure 3 for the location of these nodes). The phylogenetic reconstructions were done with the general time reversible (squares) or SRD06 (triangles) nucleotide substitution models, a relaxed clock model (uncorrelated exponential distribution of rates, solid symbols; lognormal distribution of rates, open symbols), and the Bayesian skyline as a demographical tree prior. The bars indicate the Bayesian credibility intervals (95% highest posterior density, 95% HPD) intervals estimated from the posterior distribution obtained through Markov chain Monte Carlo analyses.

The time origins of the MRCAs of isolates sampled in Madagascar and of genogroup E were estimated in 1995 (Figures 3B and 4) and showed the occurrence of recent diversification events within type EV-A71. The Madagascan isolates were directly related to genogroup B through node 1 (pp = 0.84), whose time origin was estimated in the early 1950s (Figure 4). Tree node 5 (pp = 0.91; TMRCA  = 1978) clearly showed a common origin of genogroups D and E. Although node 4 was supported by a pp of only 0.72, its occurrence in the genealogy suggested that these two genogroups shared a common origin with genogroup C in 1966 (95% HPD, 1951–1979). Finally, the MRCAs of the two major genogroups dated back to the early 1960s (genogroup B) and 1980s (genogroup C) and the tree root was estimated to the year 1924 with a credibility interval ranging between the years 1957 and 1872 (Figures 3B and 4).

## Discussion

Because of its implication in the occurrence of severe syndromes in children, EV-A71 is considered as a major human pathogen whose epidemiology and pathogenesis is now widely studied. The precise characterization and classification of clinical isolates is crucial in determining how EV-A71 lineages circulate and emerge, and in deciphering the mechanisms involved in the occurrence of severe manifestations.

In the present study, we investigated the molecular basis and evolutionary origins of the diversity among EV-A71 genogroups and subgenogroups by making a comprehensive comparison of the complete capsid protein VP1 nucleotidic sequences. Whole or partial VP1 sequences are commonly used for molecular epidemiology studies to assess the phylogenetic relationships among isolates and to classify isolates into species, types and sub-types. By targeting a capsid-encoding sequence, this usual genotyping method has led to a classification that is globally congruent with the historical classification based on serotyping methods. As EV genotyping is usually based on the VP1 capsid protein gene, it does not take into account the recombination events that occur frequently in the non-structural part of viral genomes [33], [34]. Taking the recombination features into consideration for EV genotyping would lead to a tremendously complex classification because of numerous recombinant patterns than can exist within a given type, while the consequences of recombination on the phenotypic properties of the viruses are unknown.

Both Neighbor-Joining and Bayesian analyses based on whole VP1 sequences identified six different clusters among the EV-A71 isolates. Three clusters were consistent with the ‘historical’ genogroups A-C [11]. Our analysis did not support the hypothesis that some sequences belonging to the sub-genogroups C4 could constitute a separate genogroup [35]. The analyses of the full-length VP1 sequence of a virus collected in India in 2001 [14] confirmed its assignment to a specific genogroup, designated genogroup D. During our investigation, we found that partial VP1 sequences of other isolates recently sampled in India [32] also clustered within this genogroup. These findings suggest that members of this genogroup are still actively circulating in this country. Genogroup E [15] consists of the two sequences of isolates from Central African Republic and Cameroon. Its recent identification can be explained by the low number of studies on EV-A71 epidemiology in African countries. As this genogroup was previously shown to contain an additional partial VP1 sequence from Nigeria [15], it can be supposed that members of this genogroup circulate throughout a wide region of sub-Saharan Africa. Finally, our analyses allowed the two virus isolates collected in Madagascar in 2004 and 2011 to be assigned to a phylogenetic cluster distinct from the earlier genogroups; we propose to designate this cluster as ‘genogroup F’. No previous studies of the epidemiology of EV-A71 infections in Madagascar had been made and this genogroup had therefore escaped the identification. Thus, it can be assumed that the circulation of EV-A71 in Africa and in Madagascar is probably underestimated, as is its impact on human health in this part of the world.

The origin of the isolates belonging to genogroup A and isolated in China in the 2000s and 2010s [12], [13] is puzzling, as no members of this genogroup had been isolated since the initial isolation of the BrCr prototype strain, 40 years before. In addition, phylogenetic analysis indicated a low evolutionary divergence between the Chinese isolates and the prototype strain, thereby excluding a re-emergence of genogroup A viruses after a continuous circulation for decades. The low divergence observed between the recent isolates and the BrCr strain could be due to a recent release of prototype-like viruses into the wild from either a laboratory or a naturally-preserved contaminated source. However, the hypothesis of a laboratory contamination cannot be formally excluded.

The intra-genogroup nt threshold was set at 8% in an earlier study [1] but in our investigation, nt divergence values as high as 15.4% and 16.0% were found within genogroups B and C respectively. Accordingly, unambiguous determination of subgenogroups from pairwise p-distance comparisons is difficult and needs to be confirmed by the results of phylogenetic analysis.

We considered that the phylogenetic data inferred with our sequence dataset were robust because eight phylogenetic reconstructions using different nucleotide substitution, molecular clock models, and tree priors provided estimates for the evolutionary rate parameter with wide overlapping of their Bayesian credibility intervals. Notably, the time origin of the tree root was pushed approximately 20 years back in comparison with the earlier estimate, 1941 [36]. Because the credibility interval of the TMRCA is large, the time origin of the virus still remains uncertain and may be revised in the future with the availability of additional sequences. Nonetheless, the TMRCA of genogroup B estimated in our study is in complete agreement with the previously reported time origin in the 1960s [37]. Finally, although our estimate of the TMRCA for genogroup C is close to the year 1980, the credibility interval is in line with earlier data [38]. Overall, this indicates that although the sequence dataset used in our study consists of less than 100 sequences, it is consistent for obtaining reliable data regarding the evolutionary origin of other EV-A71 genogroups.

In conclusion, our analyses show a genetic diversity of EV-A71 that is greater than generally indicated in most published reports. First, we identified a sixth genogroup, tentatively called F, that contains sequences of two virus strains sampled in Madagascar. This genogroup, suggested by preliminary Neighbor-Joining analyses performed on a small numbers of EV-A71 VP1 sequences, was confirmed both by Neighbor-Joining analyses conducted on a large dataset of sequences and by Bayesian analyses. Second, we observed an unsuspected intra-genogroup nt divergence within genogroups B and C that was as high as 16.0%. Future studies will have to evaluate the real EV-A71 genetic diversity, which is probably currently underestimated, particularly in India, where additional genogroups can be anticipated on the basis of partial VP1 sequences, and in all regions where the EV-A71 epidemiology has been poorly studied, as illustrated by the recent discovery of genogroups E and F in Africa and Madagascar.

## Supporting Information

Figure S1 Nt pairwise comparison analysis of AF119795 with VP1 sequences representative of genogroups A (in purple), B (in blue), C (in red), D (in light green), E (in orange) and F (in dark green).
(TIF)

Figure S2 Phylogenetic relationships between some Indian EV-A71 isolates and members of genogroups A to F, based on partial VP1 sequences. In the tree, the grey rectangles highlight putative additional genogroups. Below the tree, the VP1 region taken into account is shaded in grey. The percents of bootstrap replicates are indicated if higher than 90. The CV-A16 G10 sequence was introduced for correct rooting of the tree.
(TIF)

Figure S3 Comparison of the evolutionary rate estimates calculated with different models including Bayesian skyline or Gaussian Markov Random Field (GMRF) Bayesian skyride tree priors. The phylogenetic reconstructions were done with the general time reversible (GTR, square) or SRD06 (circle) nucleotide substitution model and a relaxed clock model with either an uncorrelated exponential distribution of rates (solid symbols) or a lognormal distribution (open symbols). The bars indicate the Bayesian credibility intervals or 95% highest posterior density (95% HPD) intervals estimated from Markov chain Monte Carlo of each analysis. The log marginal likelihood estimates indicated that the model indicated in red fit the sequence data better than the other models (see the results reported in Table 2).
(TIF)

Table S1 List of the 59 sequences representative of the different genogroups and sub-genogroups used to conduct evolutionary analyses.
(DOCX)

Table S2 Sequence pairs featuring extreme nt or aa divergence.
(DOCX)
