# Supplementary figures and images for: Molecular Comparison and Evolutionary Analyses of VP1 Nucleotide Sequences of New African Human Enterovirus 71 Isolates Reveal a Wide Genetic Diversity
Source: PLoS One. 2014 Mar 5;9(3):e90624. doi: 10.1371/journal.pone.0090624 (PMC3944068; doi:10.1371/journal.pone.0090624)

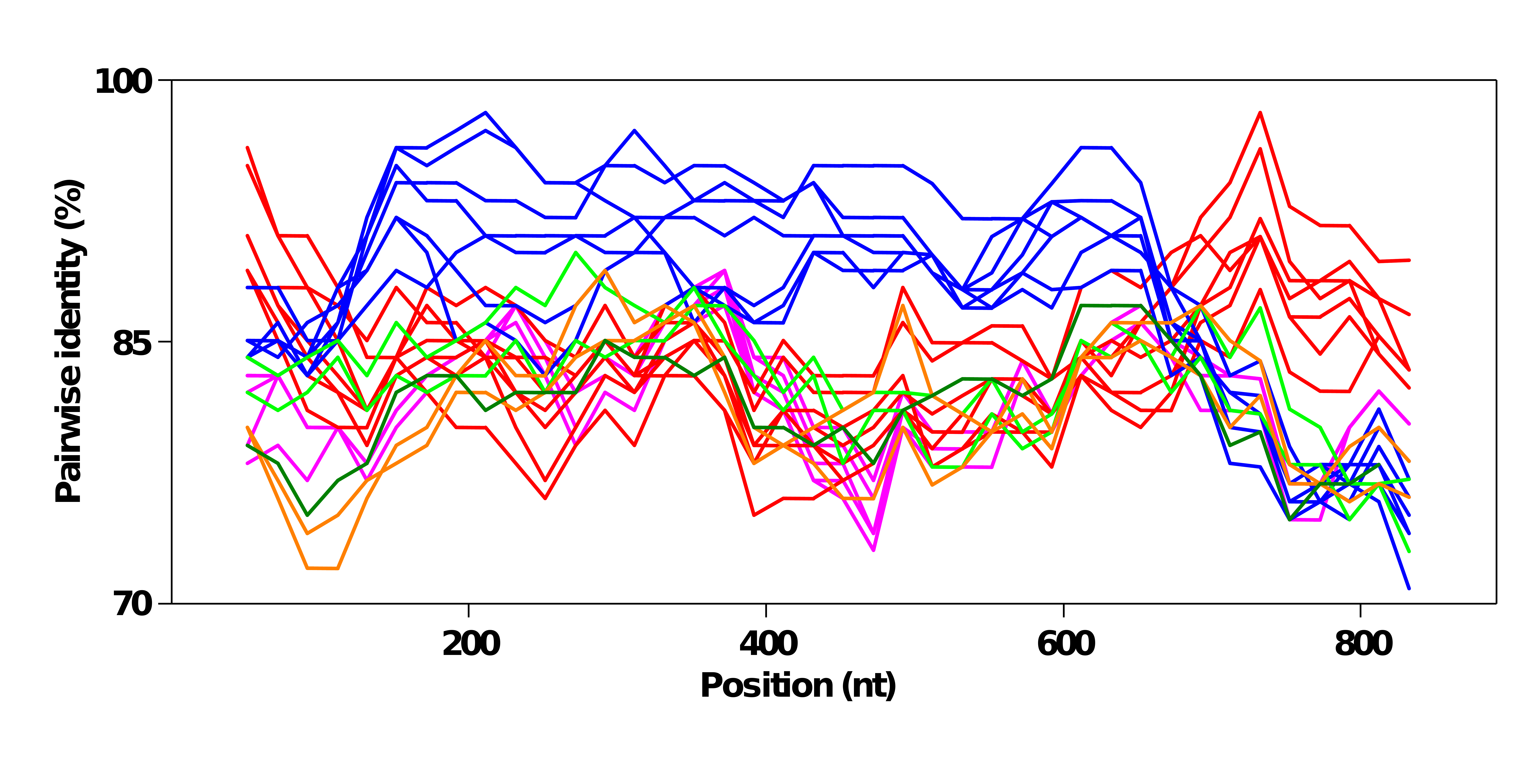

Supplement: Figure S1 — Nt pairwise comparison analysis of AF119795 with VP1 sequences representative of genogroups A (in purple), B (in blue), C (in red), D (in light green), E (in orange) and F (in dark green). (TIF) [file pone.0090624.s001.tif]

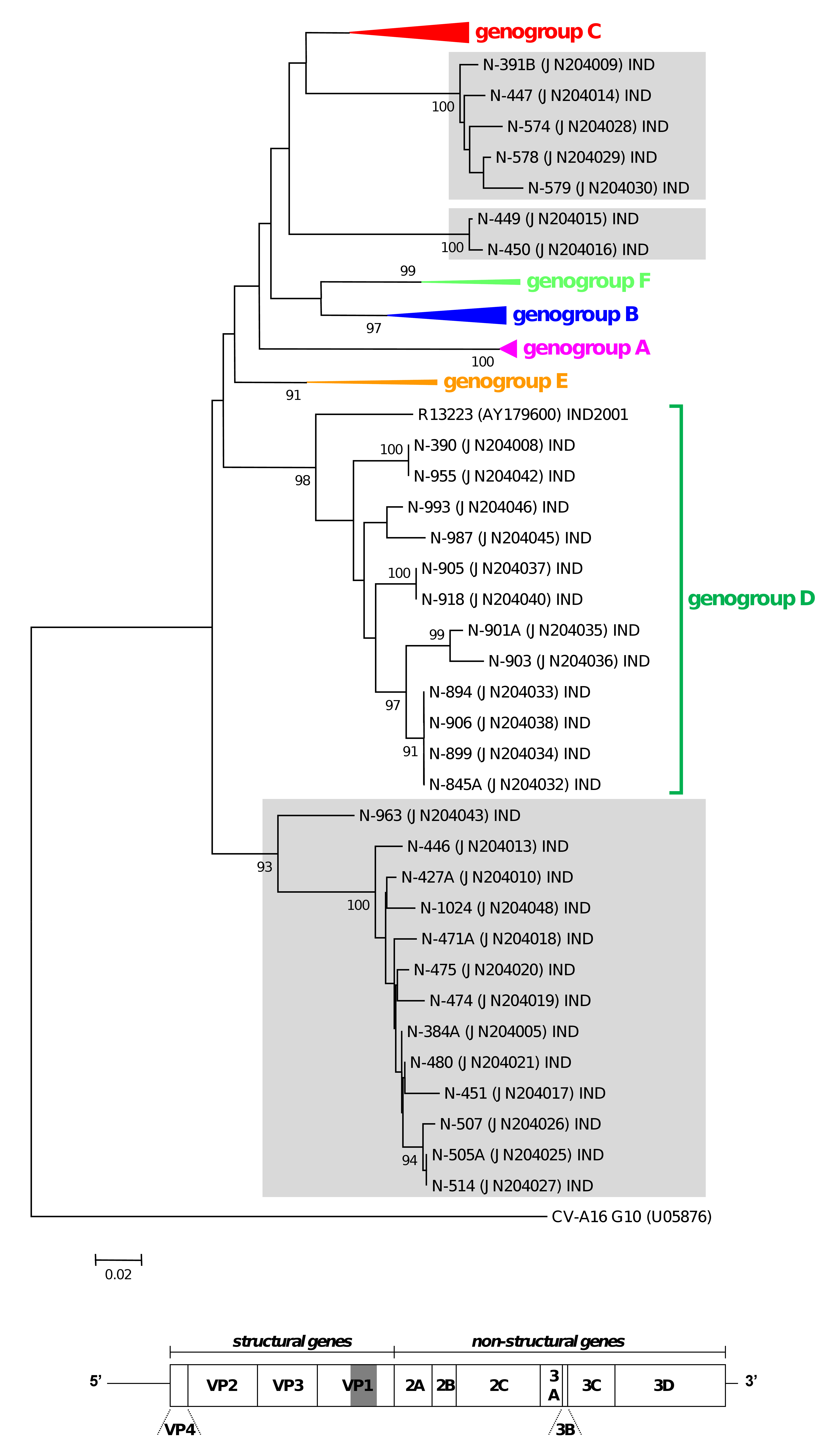

Supplement: Figure S2 — Phylogenetic relationships between some Indian EV-A71 isolates and members of genogroups A to F, based on partial VP1 sequences. In the tree, the grey rectangles highlight putative additional genogroups. Below the tree, the VP1 region taken into account is shaded in grey. The percents of bootstrap replicates are indicated if higher than 90. The CV-A16 G10 sequence was introduced for correct rooting of the tree. (TIF) [file pone.0090624.s002.tif]

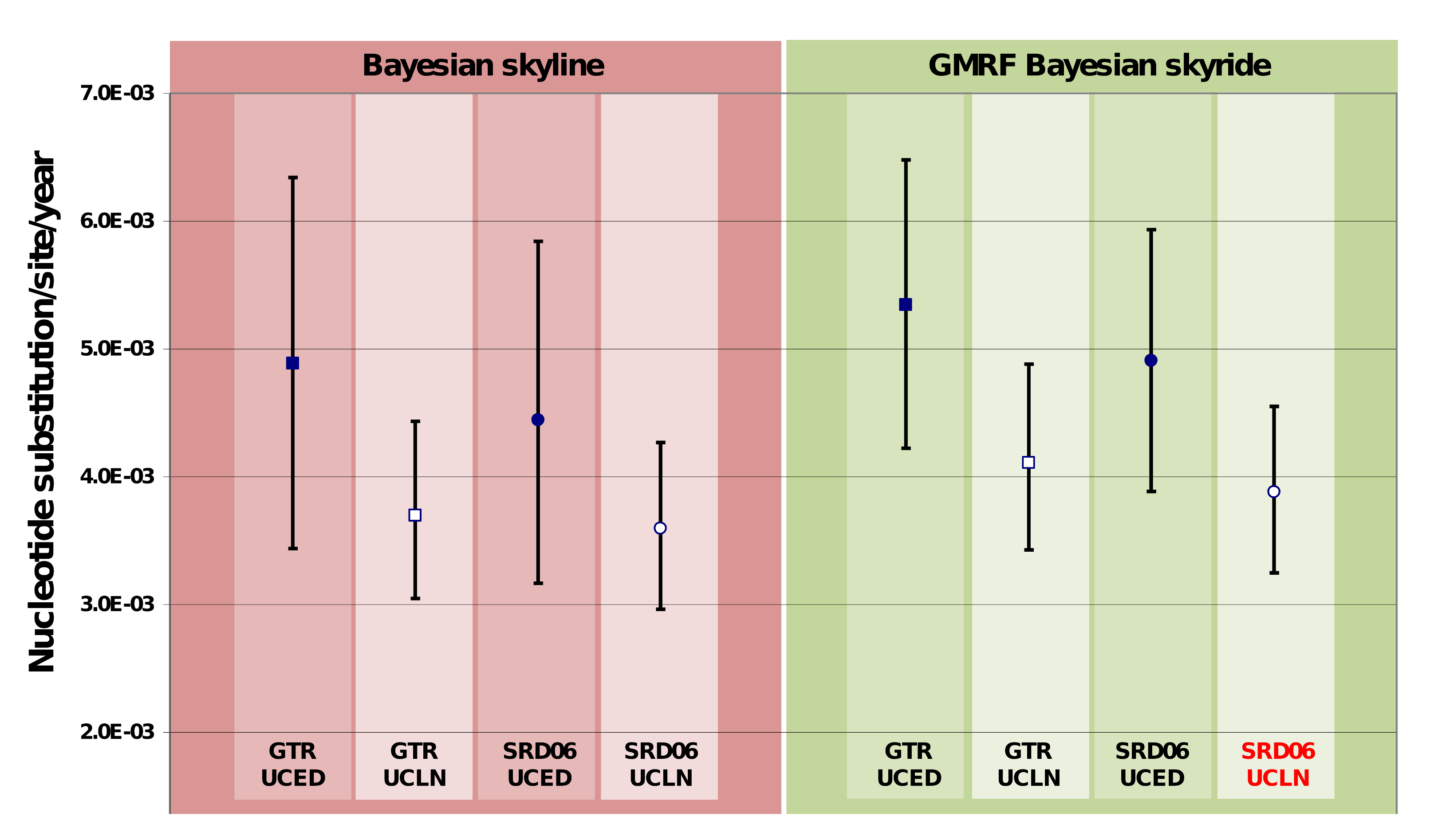

Supplement: Figure S3 — Comparison of the evolutionary rate estimates calculated with different models including Bayesian skyline or Gaussian Markov Random Field (GMRF) Bayesian skyride tree priors. The phylogenetic reconstructions were done with the general time reversible (GTR, square) or SRD06 (circle) nucleotide substitution model and a relaxed clock model with either an uncorrelated exponential distribution of rates (solid symbols) or a lognormal distribution (open symbols). The bars indicate the Bayesian credibility intervals or 95% highest posterior density (95% HPD) intervals estimated from Markov chain Monte Carlo of each analysis. The log marginal likelihood estimates indicated that the model indicated in red fit the sequence data better than the other models (see the results reported in Table 2). (TIF) [file pone.0090624.s003.tif]
