# Supplementary material for: Molecular Comparison and Evolutionary Analyses of VP1 Nucleotide Sequences of New African Human Enterovirus 71 Isolates Reveal a Wide Genetic Diversity
Source: PLoS One. 2014 Mar 5;9(3):e90624. doi: 10.1371/journal.pone.0090624 (PMC3944068; doi:10.1371/journal.pone.0090624)
Supplement: Table S1 — List of the 59 sequences representative of the different genogroups and sub-genogroups used to conduct evolutionary analyses. (DOCX) [file pone.0090624.s004.docx]

**Table S1. List of the 59 sequences representative of the different genogroups and sub-genogroups used to conduct evolutionary analyses.**

| Strain | Group | GenBank accession number |
| --- | --- | --- |
| BrCr  6984  5603  6303  11994  10745  10724  10076  10857  1834  258  6910  Nagoya  Hungary 78  7423/MS/87  3984-OH-82  11590  CHE516_GER97  04716-MAA-97  MY6/2/SAR/97  1M/AUS/3/99  SB0635/SAR/00  1141-Yagamata-00  AFP2003163-TW  5511/SIN/00  MY17/Sw/A/06  NUH0085/SIN/08  2009-03531  6887-SYD-86  THA-07-02073  Siriraj07/TH/03  CF210042_FRA06  GRE29_FRA07  CF097017_FRA03  STU546711_GER08  2286  CF1113_FRA00  CF341034_FRA06  ANG261_FRA09  97-56  03-KOR-00  06-KOR-00  3254  CF192013  ULM275  SZ/HK08-5  SZ/HK08-6  2011szk332  YN42B/YN/CHN/10  933V/VNM/05  1089T/VNM/05  2007-07364  999T/VNM/05  NTU1482-TW-06  R13223  CAF-NMA-03-008  C08-146  MAD-72341-04  MAD-3126-11 | A  B0  B0  B0  B0  B0  B0  B0  B0  B0  B1  B1  B1  B1  B2  B2  B2  B2  B3  B3  B3  B4 B4 B4  B5  B5  B5  B5  C1  C1  C1  C1  C1  C1  C1  C2  C2  C2  C2  C3  C3  C3  C4  C4  C4  C4  C4  C4  C4  C5  C5  C5  C5  C5  D  E  E  F  F | U22521  AB524090  AB491212  AB524089  AB524085  AB524083  AB524082  AB524081  AB524084  AB524086  AB059814  AF135901  AB059813  AB059815  U22522  AF009538  AB524134  FN649243  AY207636  AF376075  AF376097  AF376069  AB177811  DQ841992  AF376121  HQ676235  FJ461785  HM622390  AY722887  FJ151498  FJ846677  FN598749  FN598766  FN598734  FN649264  AF135941  FN598726  FN598750  FN598777  AB115494  DQ341356  DQ341355  AF286531  FN598741  FN649252  GQ279369  GQ279370  JQ315075  JQ264777  AM490161  AM490142  EU527983  AM490163  DQ846662  AY179600  JN255590  JX307649  HG421068  HG421068 |
