# Supplementary material for: Molecular Comparison and Evolutionary Analyses of VP1 Nucleotide Sequences of New African Human Enterovirus 71 Isolates Reveal a Wide Genetic Diversity
Source: PLoS One. 2014 Mar 5;9(3):e90624. doi: 10.1371/journal.pone.0090624 (PMC3944068; doi:10.1371/journal.pone.0090624)
Supplement: Table S2 — Sequence pairs featuring extreme nt or aa divergence. (DOCX) [file pone.0090624.s005.docx]

**Table S2. Sequence pairs featuring extreme nt or aa divergence.**

| **Sequence #1** | **Sequence #2** | **Nt divergence (%)** | **AA divergence (%)** |
| --- | --- | --- | --- |
|  |  |  |  |
| **Maximum intra-genogroup divergences** | | | |
| FJ151498 (C1) | JQ264777 (C4) | 16.0 | 4.7 |
| FJ846677 (C1) | JQ264777 (C4) | 15.6 | 5.7 |
|  |  |  |  |
| **Minimum inter-genogroup divergences** | | | |
| AB524089 (B0) | HG421069 (F) | 13.8 | 2.7 |
| AB524084 (B0) | FN649252 (C4) | 13.8 | 1.7 |
| AB524082 (B0) | AY179600 (D) | 16.8 | 0.6 |
| AB524089 (B0) | AY179600 (D) | 17.0 | 0.6 |
| AB524086 (B0) | AY179600 (D) | 16.8 | 0.6 |
| AB524090 (B0) | AY179600 (D) | 17.0 | 0.6 |
|  |  |  |  |
| **Maximum inter-genogroup divergences** | | | |
| JN255590 (E) | GQ117126 (A) | 20.2 | 5.7 |
| HG421069 (F) | GQ117126 (A) | 20.2 | 5.0 |
| JQ264777 (C4) | U22521 (A) | 19.8 | 8.1 |
|  |  |  |  |
